# The Intersection of Psychology and Gynecology: A Comprehensive Review of Pain Management Strategies in Women's Health

**DOI:** 10.7759/cureus.86344

**Published:** 2025-06-19

**Authors:** Archana Kori, Ajay Singh, Yash Satish Caroicar, Priyanka Elizabeth Thomas, Anjali Chandra

**Affiliations:** 1 Obstetrics and Gynaecology, Chhindwara Institute of Medical Sciences, Chhindwara, IND; 2 Anaesthesiology, Bundelkhand Medical College, Sagar, IND; 3 Medicine, Goa Medical College and Hospital, Bambolim, IND; 4 Nursing, All India Institute of Medical Sciences, Patna, IND; 5 Gynae Oncology and Robotic Gynaecological Surgery, Max Institute of Cancer Care, Delhi, IND

**Keywords:** biofeedback, chronic pelvic pain, cognitive-behavioral therapy, endometriosis, pain management, virtual reality therapy

## Abstract

Chronic gynecological pain conditions, including dysmenorrhea, endometriosis, vulvodynia, and chronic pelvic pain syndrome (CPPS), present significant challenges in women's health. These conditions result in significant physical discomfort and are closely associated with psychological comorbidities such as anxiety, depression, and increased central pain sensitization. Traditional pharmacological and surgical interventions often provide limited relief, as they fail to address the complex interplay between biological, neurological, and psychological factors underlying pain perception. This review explores the intersection of psychology and gynecology in pain management, emphasizing the biopsychosocial model as a comprehensive framework for understanding and treating gynecological pain. Research highlights the influence of hormonal fluctuations, central sensitization, and neuroinflammatory pathways in pain amplification, further exacerbated by adverse childhood experiences, post-traumatic stress disorder, and maladaptive coping mechanisms. Integrative approaches, including cognitive-behavioral therapy, mindfulness-based stress reduction, biofeedback, and virtual reality (VR) therapy, have demonstrated efficacy in alleviating pain intensity and improving emotional resilience. The integration of digital health innovations and personalized medicine presents new opportunities for optimizing pain management strategies. Future research should focus on interdisciplinary collaboration between gynecologists, psychologists, and pain specialists to develop patient-centered treatment modalities. A paradigm shift toward holistic and precision-based interventions is essential to enhance therapeutic outcomes and improve the quality of life for women suffering from chronic gynecological pain.

## Introduction and background

Chronic gynecological pain is a pervasive and complex issue in women’s health, affecting both physical well-being and psychosocial functioning. Conditions such as dysmenorrhea, endometriosis, vulvodynia, and chronic pelvic pain (CPP) syndrome (CPPS) are highly prevalent yet frequently underdiagnosed and undertreated, leading to persistent suffering and reduced quality of life [1,2]. Despite advancements in gynecological care, the chronic and treatment-resistant nature of these conditions reveals critical limitations in current approaches, particularly those that fail to consider the full spectrum of pain determinants [3].

While existing research acknowledges the biological underpinnings of gynecological pain, emerging evidence underscores the integral roles of psychological distress and social context. Depression, post-traumatic stress disorder (PTSD), and adverse childhood experiences (ACEs) have been closely linked to CPP [4,5], signaling a need for broader clinical frameworks. The biopsychosocial model provides a more comprehensive lens by accounting for how biological mechanisms such as hypothalamic-pituitary-adrenal (HPA) axis dysregulation and central sensitization interact with emotional and environmental stressors to amplify pain perception [6,7].

This review addresses a significant gap in the literature: the fragmentation between biomedical and psychological approaches to gynecological pain management. It aims to synthesize insights from neurobiology, gynecology, and behavioral science to highlight integrative care strategies. Specifically, it evaluates conventional pharmacological and surgical treatments [8], alongside psychological interventions such as cognitive-behavioral therapy (CBT) [9], mindfulness-based stress reduction (MBSR) [10], and digital therapeutics [11,12]. By adopting a biopsychosocial framework, this study proposes a multidisciplinary path to enhance pain management and overall well-being among women with persistent gynecological pain.

## Review

Biological and hormonal influences on pain perception

The female reproductive hormones estrogen and progesterone control the way women experience pain by affecting both peripheral and central pain signaling mechanisms [13]. The specific pain effects of estrogen include both nociceptive and antinociceptive properties that change based on its concentration and receptor activity patterns [14]. Pain perception decreases when opioidergic and serotonergic pain inhibitory pathways become more active because of high estrogen levels, but estrogen level changes create neuroinflammatory conditions that increase pain sensitivity through central sensitization [15]. Progesterone may have analgesic properties due to its role in enhancing GABAergic (gamma-aminobutyric acid) inhibition and modulating immune responses that influence pain [16].

**Figure 1:**
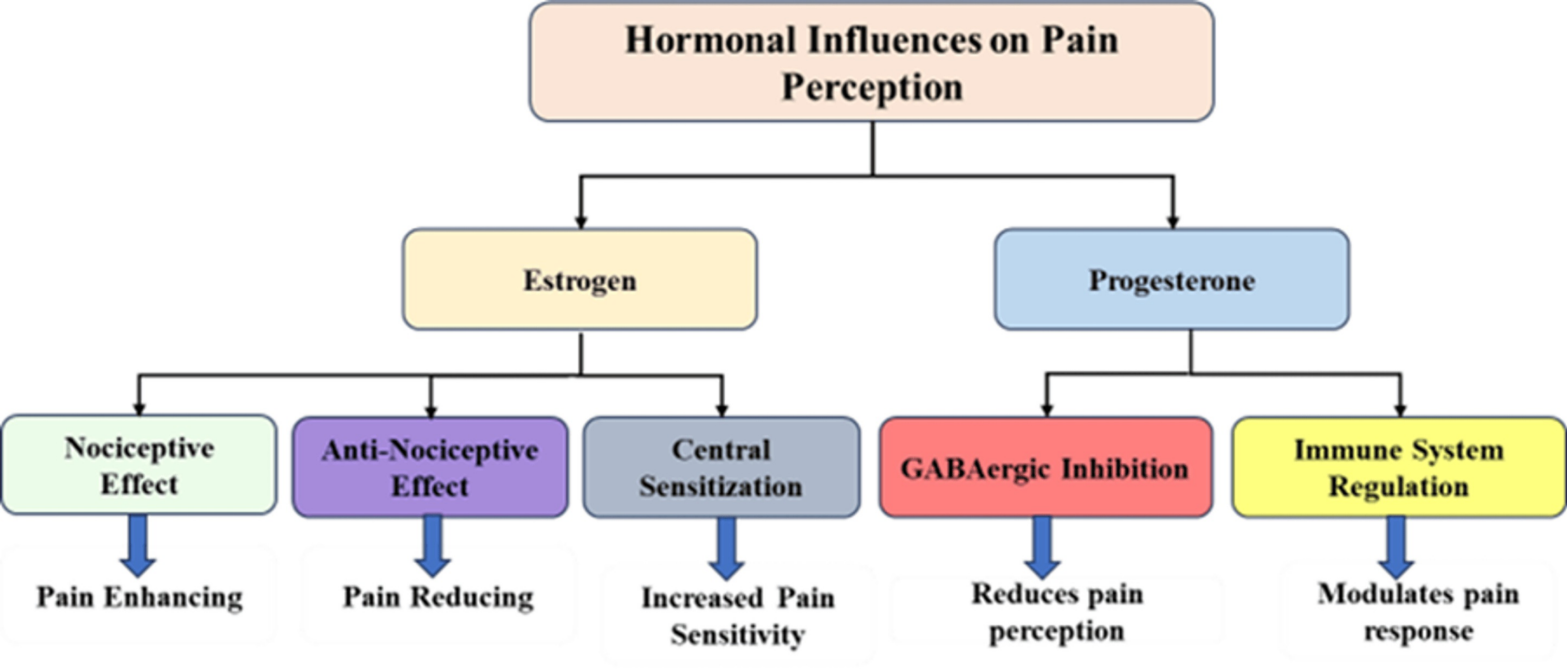
Estrogen & Progesterone Influence on Pain Perception Created by the authors

The pain perception of individuals changes substantially because of hormonal shifts that occur throughout the menstrual cycle. The luteal phase with elevated progesterone provides pain relief, yet the follicular phase containing low progesterone and changing estrogen levels creates heightened pain sensitivity [17]. The pain linked to dysmenorrhea and endometriosis gets worse during the perimenstrual period mainly because of inflammatory substances such as prostaglandins and estrogen-triggered central sensitization [18]. The pain perception of the two major hormones is illustrated in Figure 1.

Common gynecological pain disorders

Different gynecological pain disorders affect women's health heavily and their daily quality of life, thus demanding combined medical care to manage symptoms effectively. Uterine contractions triggered by elevated prostaglandins cause the pain of primary dysmenorrhea, yet secondary dysmenorrhea emerges from endometriosis along with fibroids or adenomyosis [19]. Endometriosis causes pain through ectopic endometrial tissue that initiates continuous inflammation, tissue binding, and neuropathic symptoms. CPP affects women for longer than six months and develops from multiple causes involving neuropathic systems, musculoskeletal structures, and psychological elements [20].

The neuropathic origins of vulvodynia cause persistent vulvar pain because patients have central sensitization and psychological distress [21]. The cause of dyspareunia includes vaginal atrophy together with trauma and underlying infections [22]. Women experience pregnancy-related pelvic girdle pain as well as postpartum pain because of hormonal changes, biomechanical alterations, uterine contractions, surgical trauma, and perineal injuries [23,24]. A detailed summary of all these conditions is mentioned in Table 1.

**Table 1:** Common Gynecological Pain Disorders NSAIDs: non-steroidal anti-inflammatory drugs; CPP: chronic pelvic pain; MRI: magnetic resonance imaging; CA-125: cancer antigen 125 test; CBT: cognitive-behavioral therapy

Disorder	Etiology	Symptoms	Diagnosis	Treatment	Prognosis	References
Primary dysmenorrhea	Increased prostaglandin production leads to uterine contractions	Cramping, lower abdominal pain, nausea, headache	Clinical history and ultrasound to rule out pathology	NSAIDs, hormonal contraceptives, heat therapy	Good with appropriate management	[10]
Secondary dysmenorrhea	Underlying conditions like endometriosis, fibroids, and adenomyosis	Severe menstrual pain, pelvic tenderness, heavy bleeding	Pelvic ultrasound, MRI, laparoscopy for confirmation	Treat underlying cause, hormonal therapy, surgery	Varies depending on pathology	[2]
Endometriosis-related pain	Ectopic endometrial tissue causes inflammation and adhesions	Cyclic pelvic pain, dyspareunia, infertility	Laparoscopy, MRI, CA-125 marker	Hormonal therapy, NSAIDs, surgical excision	Chronic; requires long-term management	[14]
CPP	Multifactorial: neuropathic, musculoskeletal, psychological	Persistent pelvic pain, bowel/bladder dysfunction	Comprehensive history, pelvic exam, imaging	Multidisciplinary approach, physiotherapy, CBT	Highly variable; needs multimodal therapy	[12]
Vulvodynia	Chronic vulvar pain, neuropathic origin, central sensitization	Burning, stinging, vulvar pain	Cotton-swab test, rule out infections	Topical anesthetics, nerve blocks, pelvic floor therapy	Can be managed with multidisciplinary care	[18]
Dyspareunia	Vaginal atrophy, trauma, infections	Painful penetration, vaginal tightness	Pelvic exam, hormone level assessment	Lubricants, estrogen therapy, pelvic floor therapy	Often improves with hormonal and psychological support	[8]
Pain during pregnancy	Ligamentous strain, hormonal changes, fetal nerve pressure	Pelvic/lower back pain, sciatica	Clinical assessment, ultrasound for ligament issues	Physical therapy, maternity belts	Usually resolves after delivery	[20]
Postpartum pain	Uterine contractions, perineal/cesarean trauma	Cramps, incision/perineal pain	Clinical evaluation, postpartum pain scales	NSAIDs, perineal care, physiotherapy	Typically improves in weeks to months	[6]
General gynecologic pain syndromes	Idiopathic or functional pain, often psychological components	CPP with psychological overlay	Multimodal assessment, pain scoring, psych screening	Multimodal pain therapy, psychological interventions	Varies; psychological support often key	[11]

Pharmacological therapy and hormonal interventions, physiotherapy, and CBT make up the management approach for this condition [25]. Long-term outcomes, along with pain relief optimization, require a healthcare approach that centers on patients and involves multiple disciplines of care.

Neurobiological mechanisms of pain perception

Brain-reproductive system interactions establish the complex biological processes that determine pain perception in gynecological situations. The neurological systems of the HPA axis together with the autonomic nervous system (ANS) regulate pain modulation through their response to hormonal changes and signals of stress [26]. Psychological distress leads to increased pain perception in individuals because the amygdala and anterior cingulate cortex in the limbic system process emotional and cognitive pain responses [27].

The neurophysiological mechanism of central sensitization, which affects chronic gynecological pain, results from spinal cord dorsal horn neurons becoming more active and producing ongoing pain amplification together with allodynia and hyperalgesia. Endometriosis and vulvodynia, as well as CPPS, frequently lead to this type of neuroplastic change due to extended exposure to pain signals [28]. The progression of pain becomes more persistent because neuroinflammatory pathways cause glial cell activation while releasing elevated pro-inflammatory cytokines, which boost central nervous system pain processing. Functional MRI studies show persistent abnormalities in pain-regulatory brain regions of women who suffer from CPP, according to research [29]. The development of new therapeutic approaches requires knowledge about how the brain processes gynecological pain because it helps treat the physical and mental symptoms of chronic gynecological pain.

Psychological comorbidities and pain

Gynecological pain tends to intensify because psychological co-occurring disorders like depression, anxiety, and stress strengthen the experience of pain along with reducing tolerance and disrupting natural pain regulatory mechanisms [30]. Depression and anxiety in women tend to increase their pain-related distress levels as well as functional impairment, which results in unsatisfactory treatment results [31].

Traumatic experiences, including childhood abuse and neglect, are associated with PTSD, which may alter pain processing and heighten sensitivity in chronic gynecological pain conditions. Women with PTSD experience heightened pelvic pain perception due to their PTSD-related autonomic dysregulation and hyperarousal symptoms, according to research that shows these patients have higher rates of CPP and dyspareunia [32]. The combination of cognitive distortion with catastrophizing behavior causes individuals to exaggerate their pain levels, along with feeling a lack of control over their pain, which worsens both physical symptoms and emotional responses. Catastrophizing leads to both poor treatment responses and the adoption of unhealthy coping mechanisms and disability development in women [33].

Conventional medical and surgical pain management approaches

Medical doctors, along with surgeons, lead the treatment of gynecological pain through pharmacological therapies that represent initial options for managing dysmenorrhea and endometriosis with CPP. The inflammatory effects and uterine contractions caused by prostaglandins can be effectively managed by taking non-steroidal anti-inflammatory drugs (NSAIDs) [34]. Hormonal treatments such as oral contraceptives, progestins, and gonadotropin-releasing hormone (GnRH) analogs help control hormone levels to reduce endometrial growth along with pain symptoms of endometriosis and fibroids [35]. Opioids are occasionally prescribed for severe refractory cases, but their use is limited due to risks of dependency, tolerance, and regulatory concerns [36]. Refractory cases of chronic pain receive promising treatment possibilities from new pharmacologic interventions, which include the use of neuromodulators such as antidepressants together with gabapentinoids as well as monoclonal antibodies that target inflammatory pathways [37]. The summary information about these interventions is mentioned in Table 2.

**Table 2:** Conventional Medical and Surgical Pain Management Approaches PCOS: polycystic ovary syndrome; NSAIDs: non-steroidal anti-inflammatory drugs; CBT: cognitive-behavioral therapy; TCAs: tricyclic antidepressants; SNRIs: serotonin-norepinephrine reuptake inhibitors

Treatment approach	Mechanism of action	Indications	Efficacy	Side effects	Limitations	Alternative options	Long-term outlook	Clinical recommendations	References
NSAIDs	Inhibit prostaglandin synthesis, reducing inflammation and uterine contractions	Dysmenorrhea, endometriosis, and chronic pelvic pain (CPP)	Effective for mild-to-moderate pain; limited in severe cases	GI irritation, ulcers, cardiovascular risks	Not effective for severe pain or structural conditions	Acetaminophen, topical analgesics	Safe for long-term use with monitoring for GI risks	First line for inflammatory pain management	[32]
Hormonal therapies: combined oral contraceptives (COCs), progestins, levonorgestrel-releasing intrauterine systems (LNG-IUS), and gonadotropin-releasing hormone (GnRH) agonists and antagonists	Regulate hormonal fluctuations, suppress ovulation, and reduce endometrial growth	Endometriosis, PCOS, fibroids, dysmenorrhea	Effective in reducing hormonal pain symptoms	Weight gain, mood changes, and thromboembolic risks	Breakthrough pain is possible with fluctuations	Non-hormonal pain management, dietary modifications	Effective for long-term symptom control	Recommended for hormone-related gynecological pain	[13]
Opioids	Act on opioid receptors to modulate pain signaling in the CNS	Severe chronic pain unresponsive to other treatments	Provides significant pain relief, but risk of tolerance	Dependency, respiratory depression, nausea, constipation	High addiction risk, requiring strict regulation	Non-opioid analgesics, CBT	Not recommended for long-term use due to dependency risks	Reserved for severe cases	[22]
Neuromodulators & monoclonal antibodies: TCAs, SNRIs, gabapentinoids, and biologics targeting cytokines	Modulate neurotransmitter release and immune response	Neuropathic pain, endometriosis-associated pain	Promising in chronic pain management; ongoing research	Dizziness, cognitive impairment, and immune suppression	High cost, potential immune system involvement	Physical therapy, alternative medicine	Potential long-term benefits, but requires further studies	Emerging option for chronic pain	[30]
Laparoscopic surgery	Removes ectopic endometrial lesions and adhesions	Endometriosis, pelvic adhesions, ovarian cysts	Effective in short-term symptom relief; recurrence is possible	Surgical complications, adhesion recurrence	May not prevent pain recurrence, risk of repeat surgery	Non-surgical management, lifestyle modifications	May require repeat procedures; long-term benefit varies	Considered when medical therapy fails	[5]
Pelvic nerve blocks	Interrupt pain transmission by blocking nerve signals in the pelvic region	Neuropathic pelvic pain, vulvodynia, CPP	Moderate efficacy; often used in combination with other therapies	Local irritation, temporary numbness, and nerve damage	Short-term relief may require repeated procedures	Physical therapy, neurostimulation techniques	Used as part of multimodal long-term pain management	Used when neuropathic pain dominates the clinical picture	[19]

When medical treatment proves ineffective, doctors usually suggest surgical procedures as the next step in care. The removal of endometrial lesions and pelvic adhesive tissue through laparoscopic surgery results in symptom improvement [38]. The effectiveness of hysterectomy in treating fibroids and adenomyosis comes at the expense of potential surgical side effects, along with continued pelvic pain. The adjunct treatments for neuropathic pelvic pain include pelvic nerve blocks along with radiofrequency ablation and neuromodulation procedures [39].

Psychological and behavioral pain management strategies

Behavioral and psychological approaches function as fundamental pain management tools since they address unhelpful pain processing together with emotional distress. CBT stands as a preferred method for treating CPP, vulvodynia, and endometriosis-related pain because it helps patients change harmful pain interpretations and tackle catastrophic thoughts while building stronger coping methods [40]. Research has demonstrated that CBT enhances both pain-related disability and psychological well-being, which adds value to medical treatments as an effective component [41].

Pain acceptance and emotional regulation are strengthened with the use of MBSR and acceptance and commitment therapy (ACT), which are part of mindfulness-based interventions (MBIs). The brain networks that process pain experience functional changes through MBSR, which leads to a reduction of pain intensity, while ACT modifies patients' perception of pain to decrease their psychological distress [42]. These treatment methods show particular effectiveness in endometriosis and CPP because stress, along with emotional distress, intensifies pain sensitivity. Through deep relaxation, hypnotherapy, together with guided imagery, enables the modification of pain signals that travel through central pathways in the brain. Labor pain intensity alongside pelvic pain decreases through clinical trials at levels comparable to traditional pharmaceutical treatments [43].

Biofeedback and relaxation techniques, such as pelvic floor biofeedback and progressive muscle relaxation, help patients regulate their autonomic response and neuromuscular functions to decrease the intensity of vulvodynia and CPP pain and distress [44]. The strategies offer non-invasive long-term advantages, which make them essential components of multidisciplinary pain management protocols.

Emerging integrative and complementary approaches

The emerging complementary integrative methods provide non-invasive additional treatments for gynecological pain that target biological elements, psychological aspects, and lifestyle factors. The neuromodulation pathways of acupuncture and Traditional Chinese Medicine (TCM) strengthen circulation and immune responses to help patients with dysmenorrhea, endometriosis, and CPP conditions [45]. Acupuncture proves effective in pain reduction, but TCM needs better standardized practices, which need to be confirmed by high-quality research trials [46].

Pelvic floor therapy, yoga, and physiotherapy independently contribute to enhanced neuromuscular coordination, pain reduction, and improved quality of life, reduce tension, and boost flexibility, thus providing benefits to vulvodynia patients and those with dyspareunia and musculoskeletal pelvic pain [47]. The combination of these treatment approaches with medical intervention leads to sustained pain relief according to research [48]. Polycystic ovary syndrome (PCOS) and endometriosis pain intensity can be reduced by adopting lifestyle changes that utilize anti-inflammatory diets containing omega-3 fatty acids and antioxidants, which help control hormones and immune responses [49]. These treatment methods have the potential to be effective only when patients maintain their treatment plan for an extended period.

**Figure 2:**
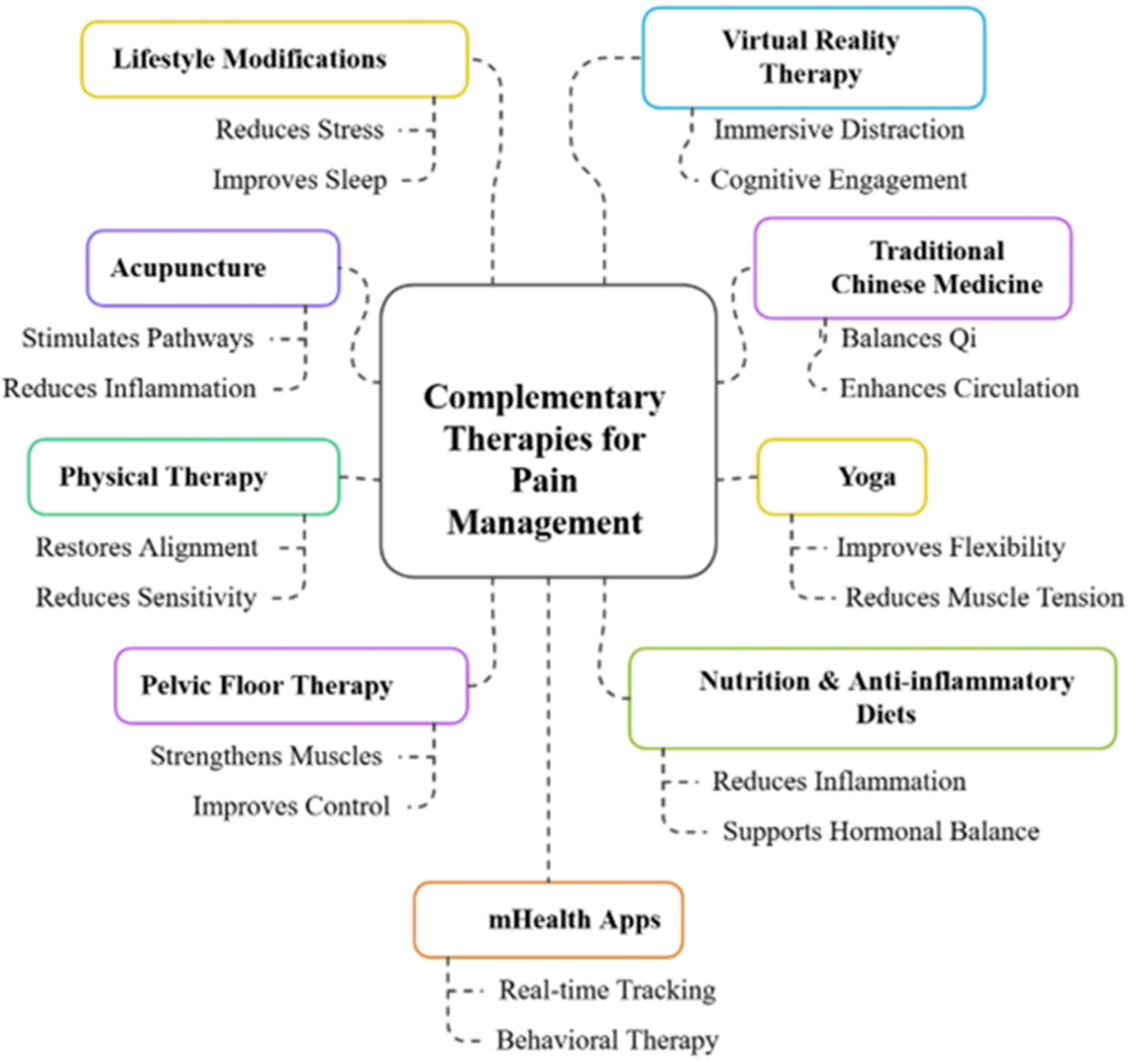
Emerging Integrative and Complementary Approaches Created by the authors

Also, medical technologies boost both patient participation and their ability to manage their condition effectively, benefiting individuals with labor pain and chronic gynecological conditions [50]. Pain management solutions from complementary therapies become more effective when used together with conventional medical treatments for gynecological disorders. The interventions that emerge as promising strategies for managing gynecology through complementary and integrative approaches are illustrated in Figure 2.

Future directions and research gaps

The progression of gynecological pain therapy depends on individual treatment plans using combined biomedical systems, psychological methods, and digital healthcare techniques. Precision medicine uses genetic, hormonal, neurobiological, and psychosocial factors to create individualized treatment approaches for patients because pain perception and treatment responses vary between individuals [51]. The discovery of pain sensitivity and treatment response biomarkers would enable medical and non-medical intervention development to maximize long-term therapeutic success.

AI and digital therapeutic advances comprise a major diagnostic and individualized treatment and real-time pain surveillance breakthrough [52]. Machine learning models powered by artificial intelligence (AI) technology processes report patient information to forecast how pain will advance and which treatments will work best for each patient, thus improving medical staff decision-making capabilities. The remote management of pain and improved patient engagement between healthcare providers and patients become possible due to wearable biosensors and mobile applications, as well as virtual reality-based pain distraction therapies [53]. Studies need to explore the long-term success and ease of accessing new approaches for different population groups.

An important challenge remains the integration of psychological care into routine gynecological treatment, which is not yet standardized in clinical guidelines. Several women with chronic gynecological pain face poor integrated medical care because gynecologists rarely work together with pain specialists or mental health professionals. Research should pursue the development of structured treatment plans that unite gynecological care with CBT and mindfulness-based approaches, along with biofeedback practice, into regular gynecological healthcare protocols.

Longitudinal research, including diverse patient cohorts, needs to become the main focus to solve present research shortcomings and eliminate healthcare equality gaps. Research findings about gynecological pain suffer from a lack of diversity in participants because most studies exclude patients from different racial backgrounds, as well as ethnically diverse and lower-income groups [54]. Future research must establish a diverse participant base to analyze how the genetic structure, combined with environmental conditions and differences in healthcare service accessibility, modifies both sensation detection and therapeutic results and patient outcomes. Long-term assessments of integrative and digital pain management strategies through follow-up studies should be conducted to verify their sustained impact and demonstrate whether they deliver meaningful improvements beyond short-lived symptom alleviation. Future developments in gynecological pain treatment will achieve better clinical results when they address essential research limitations that enable accessible and patient-focused therapies for women suffering from chronic pain disorders.

Methodological considerations

The narrative review was built using a systematic query of the PubMed, Scopus, and Google Scholar databases to retrieve peer-reviewed works published from 2000 to 2025. The used keywords and MeSH terms were chronic gynecological pain, endometriosis, vulvodynia, biopsychosocial model, psychological comorbidities, and integrative pain interventions. Only English-language studies with human female populations were searched. Relevance to biological, psychological, pharmacological, surgical, or behavioral aspects of gynecological pain was included in the criteria. The articles that were not peer-reviewed as well as those solely focused on acute pain and those that were case reports or animal studies were discarded. A total of 138 articles were identified and screened in terms of title and abstract, and 78 were fully assessed. After the assessment of eligibility, 54 studies were chosen to be included in the final synthesis. Even though it is not a systematic review, the methodology used will lead to transparency and rigor in selecting evidence as well as provide a multidisciplinary view of the management of chronic gynecological pain in women.

## Conclusions

Chronic gynecological pain conditions require a multidisciplinary approach due to psychology and gynecology working together in pain management. Hormonal changes, along with central sensitization, work together with psychological factors involving anxiety, depression, and past trauma to intensify a woman's experience of pain. Symptomatic treatment through conventional medications and surgery does not resolve the underlying psychological aspects, along with the neurobiological components of pain. The available evidence encourages the combination of CBT, MBIs, and digital therapeutics as pain management strategies. Virtual reality therapy, biofeedback, and pelvic floor therapy produce encouraging non-pharmacological strategies that help reduce pain intensity alongside improving patient quality of life. Doctors should focus research on developing customized treatments that integrate AI with precision medicine to create interventions that match patients' psychological profiles and pain systems. Future research should prioritize personalized treatment modalities, leveraging AI and precision medicine to tailor interventions based on individual pain mechanisms and psychological profiles. Healthcare organizations can enhance therapeutic results and boost patient well-being for women experiencing chronic gynecological pain by promoting interdisciplinary medical teamwork involving gynecologists, psychologists, and pain specialists.
